# Issues related to the approval of the R21/Matrix-M malaria vaccine for use in Nigeria and Ghana

**DOI:** 10.3389/fpubh.2023.1237011

**Published:** 2023-10-04

**Authors:** Kevin T. J. Dzi

**Affiliations:** ^1^Département de la formation et recherche, Institut des humanités en médecine (IHM), Centre hospitalier universitaire vaudois et Universite de Lausanne, Lausanne, Switzerland; ^2^Faculté de Biologie et de Médecine, Université de Lausanne, Lausanne, Switzerland

**Keywords:** public health, malaria, vaccine efficacy and effectiveness, malaria control and elimination, epidemic

Malaria is a potentially fatal parasitic illness prevalent in tropical areas, primarily transmitted to individuals through the bites of female Anopheles mosquitoes that are infected. Individuals in the demographic categories of infants and children below the age of 5, pregnant women, travelers, and individuals diagnosed with HIV or AIDS are more susceptible to acquiring a severe infection (1). Malaria still causes a significant number of deaths annually in low-income countries.

Over the course of time, numerous research institutions, universities, biotech companies, and pharmaceutical companies have dedicated their efforts toward developing a malaria vaccine to safeguard individuals, particularly children, residing in Africa, where the disease exhibits the highest prevalence.

The Oxford University Malaria Vaccine (R21/Matrix-M vaccine) was recently approved for use in Ghana (2) and provisionally approved for use in Nigeria (3) for malaria protection in children. Many other African countries are currently assessing the trial data of the vaccine to determine whether they will approve the vaccine to be used in their own countries. The purpose of this review is to examine some rationales for the necessity of integrating the recently approved malaria vaccine with pre-existing control strategies in Ghana and Nigeria.

The World Health Organization (WHO) first announced the availability of a vaccine against malaria caused by *Plasmodium falciparum* in 2021. This vaccine, known as RTS,S/AS01 (Mosquirix), has an efficacy of only 30%, suggesting that many children are still at risk of contracting malaria in countries where the vaccines are administered (4). Recently, a phase IIb trial of the vaccine R21/Matrix-M developed by Oxford University showed an increased level of efficacy of 77% (5). Compared to the Mosquirix vaccine, which was approved by the WHO in 2021, this is an improvement; however, it is still less effective than other standard childhood vaccines, such as those for polio (90–99% efficacy) and measles (97% efficacy) (4). Both Ghana and Nigeria have approved or provisionally approved the use of the R21/Matrix-M vaccine, recognizing its potential benefits for children. However, the vaccine's efficacy is slightly lower than that of other commonly used vaccines for children, leaving many youngsters vulnerable to infection.

I argue that, in combination with vaccination efforts, it is crucial to maintain and possibly increase the intensity of existing malaria control measures to achieve greater protection for more children and eventually the eradication of the disease in the African region. Enforcing existing control measures is a key part of the solution to preventing more children from contracting the disease, whilst the rollout of the vaccine is taking place in nations that have approved the vaccine, and deliberations are going on in other countries to determine the approval of the vaccine.

Some of the malaria control measures I propose should be reinforced, including those recommended by the Center for Disease Control that have been put in place in the past few years through the ministries of public health in different countries. This includes case management (diagnosing and treating people with malaria), the distribution of insecticide-treated nets (ITNs), indoor residual spraying (IRS), and intermittent preventive treatment of malaria in pregnant women, and infancy, amongst others (6).

In Ghana and Nigeria, their malaria control programmes have previously implemented these measures, and they should persist in reinforcing them during the implementation of the vaccine rollout. The National Malaria Strategic Plans (NMSPs) have traditionally served as the fundamental framework for setting objectives and targets related to malaria control and elimination in Nigeria, and between 2014 and 2020, they identified the measures listed above as intervention areas to reduce malaria cases in Nigeria (7). In Ghana, the distribution of ITNs was carried out on a nationwide basis in 2004. Additionally, various control measures, including the IRS and others, are presently being implemented with the aim of effectively managing the spread of malaria (8).

The enthusiasm surrounding a vaccine may result in a reduction in the reinforcement of prevention measures such as those listed above.

Given that the R21/Matrix-M vaccine has received approval in only a limited number of countries and that Africa continues to bear the highest burden of reported malaria cases globally, it is imperative to strengthen existing malaria control programmes and other preventive measures, especially in countries where the vaccine has not yet been approved. This is necessary as it may take considerable time for the vaccine to receive approval and be implemented in all African countries. In both Nigeria and Ghana, where the vaccine has obtained approval, the vaccination process for all children may face potential delays due to reported challenges in vaccine distribution within the African context. These challenges include inadequate infrastructure for vaccine storage and difficulties in accessing individuals residing in remote areas (9).

Additional measures include the provision of vaccine-related education. Historically, erroneous beliefs have frequently resulted in suboptimal vaccination programme implementation (9), demonstrating the necessity of conducting effective campaigns. One illustrative example relates to the difficulties encountered in the northern region of Nigeria in 2003, when a significant portion of the population exhibited reluctance toward accepting the polio vaccine, resulting in significantly lower rates of vaccine coverage (10).

The approval of the R21/Matrix-M vaccine in Ghana and Nigeria is a huge breakthrough in malaria control in these countries and Africa in general, but there is still a long way to go before malaria is eradicated from Africa. Apart from the fact that the vaccine's efficacy is lower than that of other childhood vaccines, it will take some time for all African nations to approve the vaccine for use in their countries and subsequently execute vaccination campaigns with optimal efficiency. In addition to embracing the vaccine, African governments must continue to prioritize additional measures of prevention and management to ensure the greatest number of their inhabitants are protected from malaria. In addition to putting these processes into action, consistent assessments and follow-ups are required to determine their effectiveness.

## Summary

To eliminate malaria worldwide, the R21/Matrix-M malaria vaccine must be used alongside current control measures.

## Author contributions

The author confirms being the sole contributor of this work and has approved it for publication.
